# The Operations of Aural Surgery

**Published:** 1910-09

**Authors:** 


**The Operations of Aural Surgery.** By C. Ernest West, F.R.C.S., and Sydney R. Scott, M.S., F.R.C.S. Pp. xii, 201. London : H. K. Lewis. 1909.

The operations described in this book are not only the ordinary ones on the meatus, tympanic membrane, ossicles, mastoid process, and those for intracranial abscess and sinus thrombosis, but also the operations upon the labyrinth, named by the authors inferior, superior and double vestibulotomy, and the operations of translabyrinthine, postlabyrinthine and lumbar drainage for meningitis, and lastly, a systematised operation for the removal of early malignant disease of the external auditory meatus and middle ear. No operation or technique is described which the authors have not themselves tested and found of value.

All the descriptions of operations are characterised by completeness of detail with respect to technique, indications, preparation, and after treatment. For this reason the book is a most excellent one for dressers, house surgeons and assistants beginning work in an aural clinic. But the book will also appeal strongly to the specialist by reason of the account given therein of the authors' experiences of the more modern operations mentioned above, which operations the writers have been so instrumental in elaborating and bringing before the profession in various publications.

